# Adolescent, caregiver and community experiences with a gender transformative, social emotional learning intervention

**DOI:** 10.1186/s12939-021-01395-5

**Published:** 2021-02-03

**Authors:** Megan Cherewick, Sarah Lebu, Christine Su, Lisa Richards, Prosper F. Njau, Ronald E. Dahl

**Affiliations:** 1 Department of Health Sciences, California State University East Bay, 25800 Carlos Bee Blvd, Hayward, CA 94542 USA; 2 Institute of Human Development, University of California Berkeley, 2121 Berkeley Way West, Berkeley, CA 94720 USA; 3 Health for a Prosperous Nation, P.O. Box 13650, Dar es Salaam, Tanzania

**Keywords:** Developmental science, Adolescence, Social emotional learning, Gender equity

## Abstract

**Background:**

Inequitable gender norms, beliefs and behaviors, are shaped by learning experiences during key developmental stages in an individual’s life course, and can have negative impacts on health and well-being outcomes. Very early adolescence represents one stage when formative learning experiences about gender inequity can have the potential to support or hinder more equitable gender norms, beliefs and behaviors. The aim of this qualitative study was to evaluate the effect of a gender transformative, social emotional learning intervention for very young adolescents (VYAs) that included experiential learning with peers, parents/caregivers and community members.

**Methods:**

This study examined the effects of an intervention designed to provide social emotional learning opportunities for adolescents ages 10–11 in Dar es Salaam, Tanzania. The qualitative sample included 279 participants. Qualitative methods included 102 in-depth interviews with VYAs, 22 focus groups with 117 VYAs, 60 in-depth interviews with parents/caregivers and 54 participant observations. A grounded theory approach was used to identify emergent themes.

**Results:**

Participants reported growth in targeted areas of social emotional mindsets and skills, including a shift in gender norms, beliefs and behaviors. VYAs reported that experiential learning in mixed gender teams provided opportunities to actively practice and reflect on gender norms, beliefs and behaviors. VYAs also reported active practice of social emotional mindsets and skills with peers, parents/caregivers and the community. Parents/caregivers reported changes in VYAs’ social emotional mindsets and skills within the home, with the community and with siblings and peers. Both adolescents and parent/caregivers reported positive change towards more equitable gender norms, beliefs and behaviors through participation in experiential learning activities and reflective discussions.

**Conclusions:**

These findings suggest that an intervention providing social and emotional experiential learning opportunities during the developmental window of very young adolescence can be effective in transforming gender norms, beliefs and behaviors. Involvement of peers, parents/caregivers and community members was effective at supporting learning social emotional mindsets and skills in VYAs. Findings encourage local and global adolescent programming to include gender transformative content paired with social emotional experiential learning with peers, family and the community and can stimulate positive change in gender norms, beliefs and behaviors to promote gender equity.

**Supplementary Information:**

The online version contains supplementary material available at 10.1186/s12939-021-01395-5.

## Background

In 2017 there were approximately 1.24 billion adolescents in the world, which represents 16% of the total global population [1]. Half of the global population of adolescents are categorized as *very young adolescents,* defined as those aged 10–14 years of age [2]. Intervention programming and research on adolescents has often focused on a wide range of ages and developmental stages of adolescence (10–19 years of age), with less focus on very young adolescents (VYAs) ages 10 to 14 years [3–5]. As a result, the unique developmental needs of very young adolescents tend to be overlooked [6, 7]. Research on very young adolescents have found that the first few years of adolescence, marked by the onset of puberty, are characterized by a period of rapid growth, adaptation, reproductive maturation and expanded cognitive processes [8–10]. Adolescent brains experience rapid growth and reorganization of neural circuitry that shape individual experiences and processing of those experiences [11]. Findings from adolescent developmental science indicate that neural responses to rewards, particularly social rewards, motivate adolescent social behaviors and emotional processing of social experiences [12].

Very young adolescents are particularly sensitive to social emotional learning experiences. This sensitivity results from a unique combination of stability and plasticity in developing neural networks of an adolescent brain and motivates adolescents to engage in highly salient social emotional learning experiences [13]. Further, research findings on adolescent social emotional programming suggests that learning experiences that are most salient to adolescents are those that include messages that harness adolescents’ deepest motives—their desire to attain respect and status in the eyes of peers or adults whose opinions they value [13]. Social emotional learning experiences shape how adolescents build their individual identities, establish behaviors, gain social knowledge and make sense of their relationships, and shape their values and beliefs [8].

During the developmental window of early adolescence, adolescents often experience heightened expectations to adhere to socially constructed norms, roles, traits, behavior, status and power that are associated with gender [14]. Gender socialization differs across cultural contexts, and are derived from collective social expectations of appropriate behaviors [15]. Harmful gender norms can drive gender inequities in the short and long-term and impact health and well-being. Globally, studies have reported that girls often face discrimination that restricts their choices, opportunities, voice, and, agency [14, 16–18]. While it is recognized that restrictive gender norms play a greater role in shaping adolescent developmental trajectories in mid to late adolescence, in many contexts, early adolescence signaled by the onset of puberty heralds a time when girls' behaviors are increasingly constrained to traditional social roles. For example, girls are often assigned domestic work in the home, lose freedom of mobility outside the house, may withdraw from school, experience greater control over female sexuality or experience coercion to enter early marriage [2, 19]. In contrast boys are more likely to be excused from domestic chores, granted more freedom outside the home, encouraged to study, to take on leadership positions and to engage in income-generating activities [19]. Harmful gender norms impact adolescents learning and experiences and research indicates that in comparison to boys, girls are more likely to have less education, enter early marriage, experience unwanted pregnancy, and are at increased risk for gender based violence [19]. Studies report that for boys, harmful gender norms can increase risk of perpetuating physical violence, substance use, HIV/AIDs and other sexually-transmitted diseases, and mental health disorders [20–22].

A study conducted with very young adolescents in Tanzania found that cultural norms around gender often result in separation of boys and girls during and after puberty, and are associated with a divergence in experiences at school, in the home and in the community [23]. In Tanzania, restrictive gender norms often result in girls being given a disproportionate share of household chores with less opportunity for mobility outside of the home or to pursue interests [7, 19]. A cultural prototype of a chaste female child is highly valued in Tanzania [24, 25]. At the same time, girls in Tanzania are at risk for sexual exploitation and transactional sex for financial gain makes girls especially vulnerable to gender-based violence, unwanted pregnancy and early marriage [26, 27]. Gender disparities in primary learning outcomes are larger in Tanzania than elsewhere in the region. Girls have lower passing rates on the primary school examination that allows entry to secondary education than boys [28]. Only a third of children that start primary school enroll in secondary school with approximately 21% of boys joining secondary schools compared to 16% of girls [29]. According to Tanzania’s Demographic Health Survey (2015–2016) adolescent girls that are out of school are five times more likely to have children, with 27% of adolescents ages 15–19 already pregnant with their first child [30]. In Tanzania, schools may force girls to undergo pregnancy tests and can expel girls who are found to be pregnant or who have given birth [28, 31].

Many schools in Tanzania are mixed-gender schools. However, schools often segregate classroom seating by gender with girls on one wide of the classroom and boys on the other which limits opportunities to learn in mixed-gender groups. Schools often include time for sports and while some games tend to be segregated by gender, some activities do allow mixed gender play and provide a positive opportunity for mixed-gender teamwork and social cooperation—an experiential opportunity to support positive gender norms and behaviors [28]. In Tanzania, social norms that drive separation of girls and boys in academic contexts, play and social activities intensifies after sexual maturation. Therefore, mixed gender activities for very young adolescents, prior to sexual maturation, represents a unique socially acceptable opportunity to encourage mixed-gender social emotional learning.

Researchers and development partners are increasingly attuned to the potential for investments in programs for very young adolescents to shape developmental trajectories in health and well-being. Programs that include gender transformative content for VYAs can be an important strategy to reduce health and well-being disparities between girls and boys in both the near and long-term [13]. One recent study conducted in India to assess the relative effect of intervening in early compared with late adolescence found that younger boys ages 13–14 years showed greater improvement in gender practices and attitudes compared to older boys ages 15–19 [32, 33]. Evidence suggests that programs that target very young adolescents can have sustaining impacts such as decreasing the spread of HIV/AIDS [34], delaying sexual debut [35], unwanted pregnancies [36], and improving a variety of health and well-being outcomes [37]. Adolescent programs that target and are tailored to very young adolescents may be particularly effective at reducing gender disparities in health and well-being.

The developmental science of adolescence can enhance intervention approaches for adolescents by matching program design and content to impactful window of opportunity in adolescent developmental trajectories. Program design can leverage findings from developmental science on the ways that rapid physical, social, emotional and cognitive changes develop in concert with changes in social roles, responsibilities and relationships to shape development. The developmental science of adolescence supports the critical importance of social emotional learning during early adolescence as adolescents seek and experiment with different roles, belief systems and behaviors [38]. Developmental science also underscores the critical importance of peers, parents and trusting relationships to reinforce social emotional learning. Effective intervention designs should be tailored to the social, cultural, economic, political and historical context to identify unique challenges and opportunities to amplify the impact of very young adolescent programs [9]. To evaluate the efficacy of developmental science informed adolescent programming to support gender equity, more qualitative research is required to identify intervention components and content that are most salient and have the greatest impact on very young adolescents.

The qualitative study reported in this article is part of the Discover Learning Intervention *(Discover)* that was launched in 2016 with the primary goal of testing a social emotional learning intervention that leverages findings from developmental science to promote gender equity among very young adolescents in the low-resource context of Tanzania. Since its inception, *Discover has* embraced a highly experimental and adaptive approach where each round of intervention was designed to leverage successes and mitigate challenges of the previous intervention implementation. *Discover* has embraced bold ideas, tested use of technology in resource-poor communities, and engaged peer, family, school, community and policy stakeholders. Of particular importance to *Discover* was the unique opportunity to support gender equity and transform gender norms, beliefs and behaviors. *Discover* targeted very young adolescents, ages 10 to 11, to leverage the sensitive maturational period before the onset of puberty. The intervention was designed to provide content on social emotional mindsets and skills woven together with gender transformative content and paired with experiential social emotional learning opportunities to actively practice learning in small, mixed gender groups.

This article describes the qualitative methods and results from the implementation of *Discover Learning*, a six-week, after-school intervention designed to introduce positive, social emotional, experiential learning. The primary aim of *Discover* is to evaluate the comparative effectiveness of a three-arm trial on social emotional mindsets and skills, and gender norms, beliefs and behaviors. A secondary aim of *Discover* is to identify intervention components with the greatest impact on outcomes. The third aim of *Discover* is to test the effectiveness of implementing the program in a low-resource setting to identify potential for reaching scale in Tanzania and to inform other programs for very young adolescents in low-resource contexts. This article seeks to evaluate the effect of *Discover* on gender norms, beliefs and behaviors and to identify the components of *Discover* that were most salient to participants and parents/caregivers.

## Methods

### Research design

#### Overview of discover

Five hundred seventy-nine very young adolescents (10–11-year-old) enrolled in *Discover* were introduced to seven adaptive social emotional mindsets and skills through viewing of Ubongo Kids video episodes on each of the social emotional mindsets and skills (Table 1). Ubongo Kids is a Tanzania-based organization that creates culturally relevant learning content for school-age children and adolescents across Africa. Ubongo Kids focuses on promoting social and behavioral change for children, adolescents and their parents/caregivers. This study curated existing animated videos from Ubongo Kids and co-created content on social emotional mindsets and skills. Prior to inclusion in the study, episodes were pilot tested on a group of 15 students between the age of 10–14 years, and reviewed by teachers, parents and representatives from the Ministry of Education in Tanzania.

**Table 1:** *Discover* social emotional learning content for very young adolescents

Social Emotional Mindsets and Skills	Description
Gender equity	Positive gender norms, beliefs and behaviors.
Teamwork	Positive peer communication, collaboration and problem-solving. Recognition of individual strengths and the benefits of teamwork.
Growth mindset	Focus on the potential for growth and development of individual abilities.
Curiosity	Encouraging motivational learning and positive risk taking.
Purpose	Exploring individual intention, engagement and prosocial reasoning to develop long-term, heart-felt goals
Persistence	Perseverance of effort and commitment to goals.
Generosity	Encouraging intentional practice of gratitude and kindness.

Study participants were randomly allocated to three intervention groups in order to determine what components of the intervention were most effective in achieving improved outcomes. At each school, an event was held where participants were invited to stand in lines outside their classrooms. A research assistant held a box with pencil sharpeners of three different colors inside. The box had a small hole large enough to fit a hand through but small enough that students could not see the pencil sharpeners. Each participant was invited to pick one sharpener at random. Participants were then assigned to Group A, B or C depending on whether they picked pink, yellow or blue pencil sharpeners respectively. A group was matched to a particular sharpener color at random. Group A participants received six sessions and watched Ubongo Kids videos on each of the seven social emotional mindsets and skills in large classrooms of up to 26 participants. Group B participants watched the Ubongo Kids videos and participated in reflective discussions in small mixed-gender groups. Within these small groups of 4–5 participants were provided with themed reflections cards to guide a group discussion. Group C received 18 sessions (3 sessions for 6 weeks) where participants watched Ubongo Kids videos paired with technology enabled experiential learning activities in small, mixed-gender groups of 4–5 participants. Each experiential learning session was facilitated by a trained young adult facilitator recruited from the local community. Facilitation principles were co-created with locally hired facilitators from the study setting [1, 2]. The objective of the principles was to guide facilitators in scaffolding positive, gender transformative, social emotional learning experiences. The six facilitation principles included: (1) scaffolding learning to create safe exploratory space for learners, (2) emphasis on learning over education, (3) withholding judgment, (4) encouraging teamwork and positive group dynamics, (5) disrupting gender norms and (6) encouraging a growth mindset. Table 2 includes a detailed description of *Discover’s* facilitation principles.

**Table 2:** The six facilitation principles used in discover learning intervention

***Principles***	Ways of demonstrating principle during instruction
***Scaffolding*** **vs.** ***Teaching*** In scaffolding a learning experience, facilitators create space for learners to safely struggle, fail, and learn from experience. This does not mean being absent or only observing the learning process, but rather providing targeting feedback at specific times. This requires providing only *essential* support and resources.	○ Provide essential information ○ Balance between success and failure ○ Build questions/encourage using resources ○ Be comfortable with struggle ○ Observe ○ Create an environment that creates positive learning experiences ○ Provide options ○ Start small when providing information ○ Everything is a learning opportunity ○ Easy tasks first, then get harder ○ Students generate ideas and give each other feedback ○ Creating a safe space to try things out ○ Model rewarding/trying ○ Practice ideas
***Learning over education*** The importance of the active process of learning –gaining knowledge or skill through experience – over the passive process of education – being taught a knowledge or skills.	Learning can mean; ○ Working towards understanding ○ There is no right or wrong ○ More freedom for the learner to explore and make mistakes ○ Do it yourself. ○ Leveraging peer understanding ○ See one do one ○ Provide immediate feedback. ○ Make learning relevant to learners ○ Experiential/hands on learning ○ Opportunity to create ○ Student-driven ○ Adaptative learning
***Non-judgment*** Learning is often a non-linear process and the learner’s path may not always be clear to the facilitator. The role of the facilitator is to withhold judgment or offering an opinion about the path. Great learning can result from mistakes and failures.	○ Accepting other people’s realities to be true ○ Being aware of unintended consequences ○ Do not generalize ○ Discourage stereotypes ○ Positive reinforcement ○ Acknowledge all input
***Teamwork and positive group dynamic*** Every member of a team brings unique skills and perspectives. It is important to create a climate where diverse skills are valued, recognized, and leveraged to help the group succeed. This includes members of the team learning to step up when their skills are most needed and relevant and step back when they are less so.	○ Use activities to get to know one another ○ Foster cooperation/collaboration among group activities ○ Create common goal/tasks ○ Use unifying factors - Group name, slogan, symbol, identity ○ Common emotional experience ○ Diversity of tasks from different people can stand out ○ Strategies for conflict resolution ○ Mix up groups - bigger, smaller, and different ○ Mediation ○ Offering options ○ Providing more structure
***Disrupting gender norms*** There are many cultural norms that affect learning. For the Discover Project a goal is to attend to these gender norms which may appear (e.g., girls stating that they are not good at a task; boys taking credit for leadership roles even when they are not the leaders; boys taking credit for girls’ actions or ideas).	○ Feeling of safety and security, where, when and with whom ○ Anyone can do anything ○ Addressing family responsibilities ○ Consider broader societal context ○ Create equal opportunities for leadership ○ Be mindful of chosen and assigned roles
***Growth mindset over Fixed mindset*** The project aims to highlight the value of taking risks and the importance of failure in learning. We will use the FAIL framework (Frequent Attempts in Learning). The more the group takes changes and fails, the more they learn. In addition, the project adheres to the approach that every individual has the potential to grow and change – no one has a finite intrinsic capacity or ability in any realm.	Having a growth mindset entails; ○ Work hard when things get tough ○ Being inspired by the accomplishments of others ○ “I can be anything” ○ Your brain is always developing ○ You are always improving ○ When you fail, try harder ○ Effort is an important part of learning ○ Try out lots of things ○ Feedback is an opportunity to learn

**Fig. 1:**
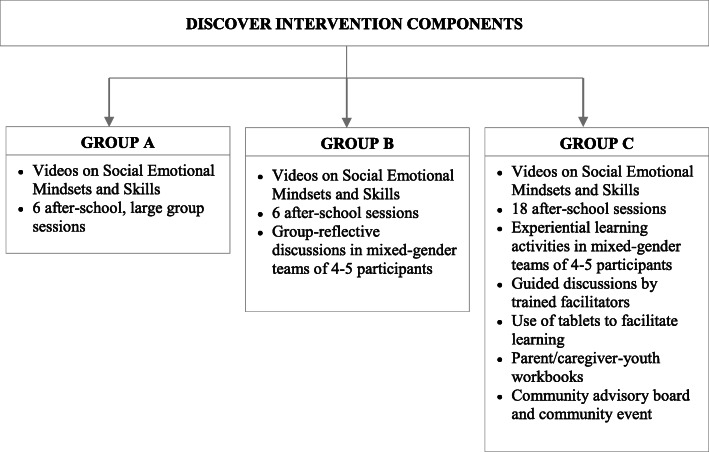
Summary of intervention components

Adolescents used tablets as an interactive tool to practice social emotional mindsets and skills in mixed-gender groups. Tablet activities included collaborative games, mind-mapping, and creative expression. For example, one collaborative activity included using tablet drawing applications to create and design a traditional batik print and proverb that could be used to create a *kanga*, a traditional Tanzanian fabric worn in a variety of ways or displayed in homes and communities that holds important cultural significance in Tanzania and in other East African communities [39]. The tablet design of the kanga was used to create actual kanga textiles which were subsequently presented to parents/caregivers and community members during a celebratory community event. The event included study participants, parents/caregivers, teachers, education officials and other important community stakeholders – to showcase the youth-led project. This event was important for communicating to parents and the community what participants had learned from the study, and to reinforce positive social norms such as encouraging parents and the community to recognize the achievements of girls and boys working together as equals. In addition to the 18 sessions, Group C participants were provided with a youth-parent/caregiver workbook that included activities and reflective discussion prompts that were matched with each of the seven social emotional mindsets and skills. Figure 1 represents a summary of the intervention components for each group.

### Study setting

This study was carried out in primary schools within the Temeke District of Dar es Salaam, Tanzania. Temeke is one of the city’s five administrative districts and is comprised of a peri-urban population. The schools that were selected included Umoja Primary School, Sokoine Primary School, Twiga Primary School and Miburani Primary School. Each school met the inclusion criteria for *Discover*; 1) government-run public schools; 2) did not participate in a social-behavioral intervention of any type 12 months before the current study; and 3), had a socio-demographic profile that matched the general school population in Dar es Salaam. The average number of students enrolled in the study schools was 956 students. The average classroom size was 63 students and each school had an average teacher: pupil ratio of 1:42. This study was conducted from July to November 2019.

### Sample

A purposive sampling frame was used to select adolescent participants in *Discover* and their parents/caregivers for participation in a qualitative study to capture the insights and experiences from adolescents and their parents/caregivers with *Discover* and the impact on gender norms, beliefs and behaviors. The criteria for selection included: full attendance in Group C of the *Discover* program; age (10–11 years); and, balance of gender representation in the qualitative study. Within the sampling frame, adolescents and parents/caregivers were randomly selected to participate in in-depth interviews and focus group discussions. Out of 579 adolescents enrolled in the *Discover* program, 102 participated in in-depth interviews and 117 took part in 22 focus group discussions. Each focus group discussion included 5–8 adolescents. Participants who participated in in-depth interviews could not participate in focus group discussions. In addition, 60 in-depth interviews were conducted among parents/caregivers and 54 observations with adolescent participants.

### Study procedures

Research activities were completed by Health for a Prosperous Nation (HPON), a local nongovernmental health research organization in Tanzania. HPON worked in collaboration with researchers from the University of California Berkeley, and actively participated in the design, development, piloting and revision of all qualitative in-depth interview, focus group and participant observation guides. The team revised interview questions to ensure cultural relevance and comprehension by adolescents ages 10–11 years. All qualitative tools were translated into the local language, Swahili. The adolescent in-depth interview guides consisted of open-ended questions related to the following topics, 1) comprehension and understanding of the key social emotional mindsets and skills, 2) experiences of learning in mixed gender peer groups, 3) experiences with the parent-youth workbook, 4) transformation of gender norms, beliefs and behaviors, and 5) experiences with using technology to learn. Parent/caregiver interview guides consisted of open-ended related to the following topics, 1) experiences of their children during *Discover,* including with the use of technology to learn, 2) how well *Discover* was implemented, 3) observable changes in their children’s behavior, and 4) experiences with the parent-youth workbook. The adolescent focus group guide included the following topics, 1) comprehension of *Discover* social emotional mindsets and skills, 2) working in mixed-gender teams, and 3) experience with using technology in learning. Probes were developed with the Tanzanian research team to capture greater depth in participant responses. Lastly, participant observation data was collected at each of 12 sessions. Participant observations focused on collecting data on how well *Discove*r implemented facilitation principles and observational data on adolescent engagement with peers and facilitators. After final revisions to the qualitative guides, the local research team participated in a 14-day training in administration of consent, human subjects research ethics, qualitative interview skills, focus group methodology and participant observation methodology.

Prior to the inception of *Discover,* HPON and the University of California Berkeley led the formation of a national advisory board (NAB) and a community advisory board (CAB) that included representatives from the Ministry of Education, Ministry of Health, members of the local government, international and Tanzanian non-governmental organizations, teachers and community members. The community advisory board provided valuable input and feedback on the design of *Discover.* With the CAB, *Discover* developed a protocol for referring participants who became distressed during in-depth interviews and focus groups to local support services. Support services were provided by a representative appointed by the CAB and participants were provided with a card with printed contact information to report any discomfort or adverse experiences. In addition, during implementation of the intervention, the study provided a community “drop box” where students could report questions, concerns and provide feedback during the intervention. Additional key partnerships included Save the Children, Dahlberg Consulting, Ubongo Kids and Camara Education. This study was approved by the Committee for Protection of Human Subjects Institutional Review Board at University of California Berkeley in July 2019. The primary local partner, Health for a Prosperous Nation obtained ethical clearance for research activities from the Tanzania National Institute of Medical Research in May 2019. Informal approval and endorsements were obtained from Temeke Municipal Council and the Ministry of Education in Tanzania prior to implementation of the study. At the conclusion of the study, participating schools were gifted technology gadgets and *Discover* learning content to enable continuance of the intervention.

### Data collection

All data collection activities were completed by HPON, local Tanzanian health research partners. Parents/caregivers of adolescent participants were provided with information pertaining to the purpose of the qualitative study, risks and benefits of participation and were asked to provide informed consent for their own participation and informed consent for their adolescent’s participation. If parents/caregivers gave consent for adolescents to participate in the qualitative study, their adolescent was provided with the purpose of the study, risks and benefits of participation, and was asked to provide verbal assent to participate in the study. No participant names were recorded and unique identification numbers were assigned to all participants. After the Tanzanian research team obtained consent and assent, trained interviewers selected a location for the interview or focus group that would allow for privacy and disclosure. All interviews were conducted in private and no information was shared outside the research team. All in-depth interviews and focus groups were conducted in Swahili and were audio-recorded. Translation was completed by a professional translator from Swahili to English. Interviewers wrote field notes during each interview to complement the audio-taped responses. The field notes were examined with the translated transcripts for important contextual interpretation of data which may have otherwise been lost in translation. The field notes also explored impressions, behaviors and nonverbal cues that the interviewer thought important to the study. The translations were cross-checked by the Tanzanian research team and discussed with researchers at the University of California Berkeley.

Interviews and focus groups began with open-ended questions designed to establish rapport so that participants felt comfortable answering questions. Interviewers used probes to explore topics in greater depth. In-depth interviews lasted 45–60 min in length. Each focus group discussion was comprised of 4–8 adolescent participants and included a facilitator and note-taker that lasted 45–60 min in length. Parents/caregivers were provided compensation for their time equal to 5USD, in the form of mobile phone airtime credits. Adolescents received a notebook and pencil as compensation for their time estimated to be valued at less than 2USD. This compensation was considered appropriate after consultation with the research team, teachers, and parents/community members. Trained observers conducted 54 participants observations to assess the implementation of facilitation principles during experiential learning activities. Observers rated facilitators on each principle ranging from 1 to 5 (1 = *Not at all*; 5 = *Very well*). Data collection was completed in the months of October and November 2019.

### Data analysis

Each transcript was identified through the assigned unique identifier and entered into Atlas.ti [40, 41]. A grounded theory approach was used to identify emergent themes [42, 43]. This method of data analysis was selected because it is based on a constructivist epistemology which recognizes that meaning is co-created through discourse within a particular cultural context. The analytical steps were conducted by two separate analysts. *Line-by-line* coding of each transcript was used to identify *initial codes* that captured implicit meanings, and common relationships and significance between codes [42]. Where appropriate codes were left in vivo to preserve participant language and meaning. Next, *focused codes* were developed from the most significant and/or frequent *initial codes* to make analytical sense of the data. *Axial coding* was used to represent the content of focused codes and to connect relationships between codes, categories and concepts. During the application of focused and axial coding, analysts met to discuss and revise codes until consensus was reached. Final coding structure was applied to each transcript using Atlas. Ti software. Researchers wrote memos to capture connections and to construct analytic notes. Researchers held discussions to help identify emergent themes and connections between themes through an iterative deductive process [42, 44]. Exemplar quotes were selected to illustrate emergent themes. We attempted to control for interpretation bias by having two separate analysts interpret the data. Intercoder agreement checks were carried out on each transcript. After each check, the analysts discussed coding discrepancies for all codes applied. Any discrepancies were resolved to develop an initial codebook. The thematic codebook was revised as necessary to reflect any changes to code definitions. Further refinement was completed after the focus group transcripts and observation notes were coded.

## Results

Sample demographics are shown in Table 3. The final adolescent sample included 117 participants in focus groups and 102 in-depth interviews. Sixty parents/caregivers completed in-depth interviews, 25% of whom were male and 75% female. The ages of parents/caregivers ranged from 19 to 66 with the mean age 37.8. Fifty-four participant observations were conducted across 12 sessions. Results from participants observations on a scale of 1–5, with 1 = *not at all* and 5 = *very well*, found the average rating across all six facilitation principles was 4.8.

**Table 3:** Descriptive summary of all qualitative data collected in the study

	Total	Male	Female	Age
	N	n (%)	n (%)	Mean (SD)
Youth focus group discussions	117	58 (49.6)	59 (50.4)	10.4 (0.5)
Youth in-depth interviews	102	49 (48.0)	53 (52.0)	10.6 (0.5)
Parent in-depth interviews	60	15 (25.0)	45 (75.0)	37.8 (9.4)
Participant observations	54 observations across 12 sessions			

Thematic analysis of in-depth interviews and focus group discussions uncovered four main themes: (1) Transformation of gender norms, beliefs and behaviors; (2) Novelty, motivational learning and mastery with technology; (3) Extending learning, practice and reflection outside the classroom; and (4) Demonstrating social emotional mindsets and skills. Table 4 shows the emergent themes and representative quotes from the study.

**Table 4:** Emergent themes and representative quotes by qualitative data collection method

Emergent Themes	Youth in-depth interviews	Focus groups	Participant observations	Parent in-depth interviews
Novelty, motivational learning, and mastery with technology	“At first I did not know some parts of the tablet but now I know them and will not face a challenge when I grow up.” “Using the tablet was good; it (tablet) simplifies work and through it work can be accomplished within a short time” “We did not know how to use it (tablet) and we were instructed, and we now know how to use it. This will help me; if I have a job in the office, I will do it using a tablet.”	“I loved being able to use the tablet, to play games and to create different things.” “The things which I have learned in the tablet include playing games, drawing Batiki and I also wrote about the things which I like.”	“I noticed moments like during drawing maps where participants were so excited to show what they had drawn. For example, when the facilitator asked who wants to come in front and show us their map? Almost all the ones who had finished raised their hands. This shows they were enjoying learning new things.” “They seem to enjoy working with the tablets and most of the youth were very confident using the tablets.”	“Currently the world is globalized. If you know how to use the phone, you can see and learn many things. You can even receive a degree by using the phone. I see that it has been good for her (daughter) to learn through this tablet and the videos which they have been watching by using the projector … children understand more if you teach them through practical ways.” “There is a computer there at home so now days I see her playing with it after she completes her tasks. This has made me to know that she has learned a lot.”
Extending learning, practice and reflection with peers, family, and community	“I told them (my family) that in the project, I had learned about setting goals and I asked them what their goals are. They told me that they don’t know their goals. I asked them to select if they want to be teachers, police or anybody else and they started to choose their goals. After I had finished explaining it to them, they said that they now know their goals.” “I felt good and comfortable, even if I didn’t know how to answer the question, she (mother) directed me. She did not give me the answers but directed me on how to get the answer. If I missed it, she would direct me several times until I got the right answer.” “What made me happy was to draw a family map. I was also happy when I did different tasks with my father or my mother.”	“Batiki was meaningful to me because we can make Batiki and it can bring development to the community. They can also teach people to read words which are written on the Batiki. Once they read them, it can bring unity and love within the community.” “I used to do the activities with my father as my mother was telling me that she is sick or she continues with her business outside. I used to do the work with my father at night when he comes back (home).” “Today before I came to school, I went to put on my shoes and clothes and I started to sweep. My grandmother told me not to sweep and that my sister will come to sweep. I told her that work does not choose gender. I can also wash dishes and cook.”	*Not applicable*	“Before the project our relationship was very weak but after we started to do exercises together from the work book, we are now very close and I normally inspect her books to see what he has learned. The work book has motivated me to make follow up on his studies.” “I also learned something on gender equality. There are some tasks which I was supposed to help my wife do, but at first, I used to leave her to do all those tasks alone. I have now started to participate in doing those tasks like sweeping our surrounding environment and arranging items inside the house. I used to see all these jobs as the responsibility of women only but I discovered that I need also to help my wife.”
Transformation of gender norms, beliefs and behaviors.	“I explained to them that there is no special work for boys and girls; a boy can cook and a girl can do carpentry.” “It was difficult at first because we used not to care about gender balance. We used to stay separately, girls on their side and boys on their side but the facilitator insisted us to consider gender balance, boys and girls should sit together.” “We used even to be afraid of going to ask girls questions but now we are asking each other questions.”	“I liked the song that says, ‘Who said there are works for girls and works for boys, this is not right.’ It reminds me to collaborate with boys.” “I loved the lessons we were taught in the classroom and I also loved many things like playing with the tablet and drawing Batiki” “I liked the things which we did. We all collaborated in groups without discrimination, one person was answering a question, and another one was writing while the other one was also answering questions, that was how teacher told us in the group.”	“In drawing a map all boys were fast to finish drawing before girls but girls participated lastly as they seemed to think a lot of what they liked before they drew, and lastly all of them went in front one by one to show and explain what they had drawn.” “Both girls and boys worked together in all activities. For example, both participated in answering questions, during activity of sandwich making, both worked together since a boy made a sandwich for a girl and a girl made one for a boy as well; also, during drawing both boys and girls worked together.”	“I think he has been influenced, for example, the way they used to stay in groups, the way they used to discuss and help each other in the tasks given without regarding if the the task belongs to girls or boys. I think he has been influenced from these trainings. They collaborated in all the tasks, boys and girls.” “When she (daughter) comes home (from school), there is a lot of work to be done but it is not like she is going to do everything, no! She needs to have time to study. She needs to have time to learn about different things so that there are same opportunities for boys and girls. That was very interesting to me.”
Demonstrating social emotional mindsets and skills.	“We used to quarrel in choosing the right answer for the questions given but I advised my colleagues not to do that. We should instead listen to each one’s answer, put them together to get one answer.” “A creative person is a person who creates things, he or she thinks about something which no one has ever thought about and he/she creates it. You can say that those who use tailoring machines are creative; they have simplified the work of tailoring clothes.”	“The important thing which I have learned in Discover Learning is persistence. If you fail in the exams and you become persistent, you can later on pass the exam.” “The importance of generosity is that, if a person has a problem, you can help him or her by comforting or encouraging him or her or by giving him or her anything. Also, the importance of team work is to collaborate without discrimination.”	“It was hard to use one laptop since everyone wanted to sit near the laptop so they had to sit in any how so long as everyone to watch the video, but they had conflict with another since no one wanted to stay far. Another conflict was the time of drawing the symbol, everyone wanted to be the leader to lead others on how to draw, in that way it brought a conflict.” “The youth respond to the session positively; they were happy and participated well. For example, writing their purpose and presenting it in front of others was an exciting activity.”	“My child has been so much curious. She can ask you complex questions. One day she came and asked me what menstruation was. I failed to give her the answer. Now days she has a lot of questions. She is now more curious than she used to be.” “I started to observe initial changes because he used not to be able to stand in front of the teacher and read a book, but now days he has confidence, even if he wants to do something, he will do it with confidence.” “Before the project, she just used to cry whenever she faced any problem but now days when this happens, she can solve them. She follows her friends and adults for help.”

### Transformation of gender norms, beliefs and behaviors

The most commonly reported learning experiences in *Discover* were the novel experiences of working in small mixed gender teams. Adolescents reflected on how this set of experiences was different from what they were used to in a traditional classroom where seating assignments are segregated by gender. Importantly, both boys and girls reflected on how working in mixed gender groups was a positive aspect of *Discover* and an experience that they found striking.

“We don’t discriminate each other; at first we used to sit girls on our side and boys on their side, but Discover has helped us until today we love each other.” (Girl, 11, 4^th^ Grade)

“We used even to be afraid of going to ask ladies questions but now we are asking each other questions.” (Boy, 10, 4^th^ Grade)

“I have learned to collaborate with boys, at past I used not to like to sit with boys.”

(Girl, 11, Group C, 4th Grade)

“I used not to like to sit with girls and when my friends saw me with girls, they used to call me a gay something which made me not to like sitting with girls, I was also fighting with girls when they provoke me. When this project came, I started to hear about gender balance and we were taught that there are no special works for boys or for girls only. From there I started to play with girls, to wash dishes and do other works also.” (Boy, 11, Group C, 4th Grade)

Participants reported the importance of working together to learn and to solve problems including day-to-day real-life challenges and the equal value of both boys and girls in finding solutions to problems. For example, participants described that they had been competitive with peers in the past and resistant to helping one another, but after their experience with *Discover* they embraced cooperation and collaboration.

“We used to quarrel in choosing the right answer for the questions given but I advised my colleagues not to do that. We should instead listen to each one’s answer, put them together and compare them to get one good answer.” (Boy, 10, 3^rd^ Grade)

“To work in a team, like here in class we sit in groups and start to discuss and read, if there is something which you don’t know, you ask your colleague to direct you and if he/she doesn’t know something, I also direct him or her and we all understand.” (Girl, 11, 5^th^ Grade)

Learning in mixed gender teams challenged normative gender roles, beliefs and behaviors. In addition to gender transformative content, trained facilitators were able to guide groups in reflective discussions or during activities to identify gender inequities and suggest more equitable mindsets and behaviors. The active practice and reflection on gender norms, beliefs and behaviors extended outside of the classroom as adolescents described changes in their mindsets and behaviors during regular social play with peers in the community. Participants described practicing more equitable gender behaviors such as completing chores in the home typically ascribed to be completed by one gender over the other. Moreover, adolescents described motivation to share learning around gender with siblings, friends and community members.

“I told them [Parents] that we need to collaborate to do works at home and I also told them that all works are equal; there are no works which are special for boys only or girls only.” (Boy, 11, 5^th^ Grade)

“Today before I came to school, I went to put on my shoes and clothes and I started to sweep, my grandmother told me not to sweep and my sister will come to sweep, I told her that works does not choose gender, I can also wash dishes and cook.” (Boy, 10, 3^rd^ Grade)

Parents described examples of changes in their children’s beliefs, attitudes and behaviors around gender. For example, many participants described the importance of gender equity in job aspirations. In particular, girls reported that they felt new agency to aspire to take on jobs in male-dominated industries such as law and engineering. Participants also demonstrated and practiced their shifting beliefs around gender. The most common example was that household chores were no longer viewed as chores separate for girls and for boys. As a result, many participants self-initiated taking on chores that were previously reserved for the opposite gender. Importantly, many adolescents were able to vocalize their changing beliefs and attitudes surrounding gender equity to their peers, siblings and parents. Parents themselves described the effect these discussions had on their own beliefs.

“There are some tasks which I was supposed to help my wife do, but at first, I used to leave her to do all those tasks alone. I have now started to participate in doing those tasks like sweeping our surrounding environment and arranging items inside the house. I used to see all these jobs as the responsibility of women only, but I discovered that I need also to help my wife. Therefore, gender balance was a good exercise.” (Father, 34, Business owner)

“I can now see that male and female children can work together.” (Sister, 28, unemployed)

“The aspect of gender equality helps to bring equality because when she comes home there is a lot of work so it is not like she can do everything! She needs to have time to study. She needs to have time to learn about different things so that there are similar opportunities for boys and girls. That was very interesting to me.” (Father, 26, Entrepreneur)

### Novelty, motivational learning and mastery experiences with technology

Use of tablet technology in *Discover* provided a novel experience for adolescents. While many participants were familiar with Ubongo Kids videos, it was a unique experience to view visual content on social emotional mindsets and skills in a mixed-gender, social setting. Participants named characters from the videos and the associated a learning construct. For example, for the episode on gender equality, participants recalled the song about boys and girls being at liberty to do any job. Such connections were easy to conceptualize, memorize and share with others.

Interviewee: “In those videos of Discover, Kibena wanted to learn about carpentry though she was a lady, Koba also wanted to learn about cookery though he is a boy. Their teacher prohibited them; Kibena wanted to make a chair, she was refused to do so but she did not hear. One day they were passing along the way, they smelled a good flavor of pilau and when they went there, they found Koba cooking; they asked him why is he cooking while he is a boy? He told them that he learned from his mother, his mother taught him how to cook. They told him that carpentry is the work for boys, but he told them that even boys can cook pilau.”

Interviewer: “What do you think you have learned from these videos?”

Interviewee: “I have learned that there is no need for gender segregation and that woman and men can work together.” (Boy, 11, 4^th^ Grade)

“Currently the world is globalized. If you know how to use the phone, you can see and learn many things. You can even receive a degree by using the phone. I see that it has been good for her to learn through this tablet and the videos which they have been watching by using the projector...children understand more if you teach them through practicals.” (Father, 36, Mechanic)

Learning with technology encouraged risk taking, persistence and reinforced a growth mindset. For example, *Discover* activities often required the practice of persisting with multiple attempts to complete a task (repeatedly failing and trying again).

“I have learned to repeat something several times when I make a mistake until I know how to make it. I have also learned to be persistent.” (Girl, 10, 4^th^ Grade)

“I have learned not to give up even if you lose you have to try more and more.” (Boy, 11, 5^th^ Grade)

Practicing persistence to obtain mastery of a skill frequently benefitted from collaboration and sharing with peers. At the beginning of the project, most participants had limited exposure to technology. Learning to use technology was a novel experience and enhanced motivational learning. One of the first activities of *Discover* included a simple orientation to tablets, and several participants reported that one of their most memorable and exciting achievements working with a tablet was simply learning to turn it on and off. Over the six-week intervention, participants were able to reflect on their growth in technology skills and tie these experiences to the social emotional mindsets and skills included in the content of *Discover*. Facilitators were able to help scaffold participant experiences of mastery.

“We first learnt how to switch it [the tablet] on and off. We did not also know how to draw, and we used to make errors and erase it to the extent that we [team] used to finish the last, we did not give up until we knew how to.” (Girl, 10, 4^th^ Grade)

Interviewer: “Tell me about an interesting thing which you did in the tablet.”

Interviewee: “What interested me were the Batiki’s designs.”

Interviewer: “Why?”

Interviewee: “Because we made errors in most of the designs and we tried to erase them until we mastered how to do it.” (Girl 10, 4^th^ Grade)

### Extending learning, practice and reflection outside the classroom

Many adolescents mentioned that they discussed *Discover* content and experiences with their parents/caregivers, siblings and friends who were not part *Discover* participants. Learning was reinforced across different contexts and relationships. In particular, parent/caregiver involvement with *Discover* and the Parent/caregiver workbook allowed parents to reinforce social emotional mindsets and skills in the home.

“I felt good and comfortable, even if I don’t know how to answer the question, she [mother] directs me, she does not give me the answer but directs me on how to get the answer, if I miss it, she will direct me several times until I get the right answer.” (Girl, 11, 4^th^ Grade)

Many participants demonstrated a desire to use what they had learned to help and teach others. For example, participants described sharing lessons about generosity with parents and friends.

“Generosity is all about helping fellows when they experience a problem, to help my parents and to respect all other people. They will love you.” (Girl, 10, 4^th^ Grade)

“She (mother) used the workbook to ask me questions like “What does being generous help you with? And I told her that generosity is to help a person so that another day he may also help you.” (Boy, 11, 5^th^ Grade)

Parents reported observation of positive changes in their adolescent’s peer relationships and with siblings and extended family members.

“Working in collaboration within the groups has made her not to be afraid even of asking questions. It has helped her to know that she has the right to be listened to and even if she is wrong, she will be corrected, and this is a way of learning.” (Mother, 35, Entrepreneur)

“I have witnessed him resolving a fight between two of my neighbors’ children. He asked them why they were fighting instead of taking books and reading. He asked them to go with him so that he could show them what he had learnt at school.” (Mother, 40, self-employed)

An emergent theme in parent/caregiver in-depth interviews was the opportunities for parents/caregivers to actively reinforce learning through use of the parent/caregiver-youth workbook. Parents described improved relationships with their child and these reflections were provided by mothers, fathers and extended family members.

“The relationship with my grandchild has been closer because of that book. There are many things which are discussed there like the issue of collaboration, love etc. Our closeness has increased because of that book.” (Grandmother, Age unknown, unemployed)

“First, we have become friends because at first, we used to fear one another. She used to come and ask me to teach her and I direct her to her older siblings. Due to the nature of our jobs, we normally come home late and very tired and don’t like to be bothered by questions. But she told me that she was told that I should also pass through that book, we should do this and learn together.” (Father, 36, Mechanic)

Parents noted additional value of the workbook as a record of learning to return to and reference after completion of *Discover*.

“The workbook can be used to store records to remember your goals which you set, the goals which you spoke in words and wrote down. It also becomes like a reminder to a child so that when she goes astray you remind her about her goals which she had set and tell her that she needs to achieve those goals. The book will act as a reminder tool for a child.” (Father, 34, Business Owner)

**Fig. 2:**
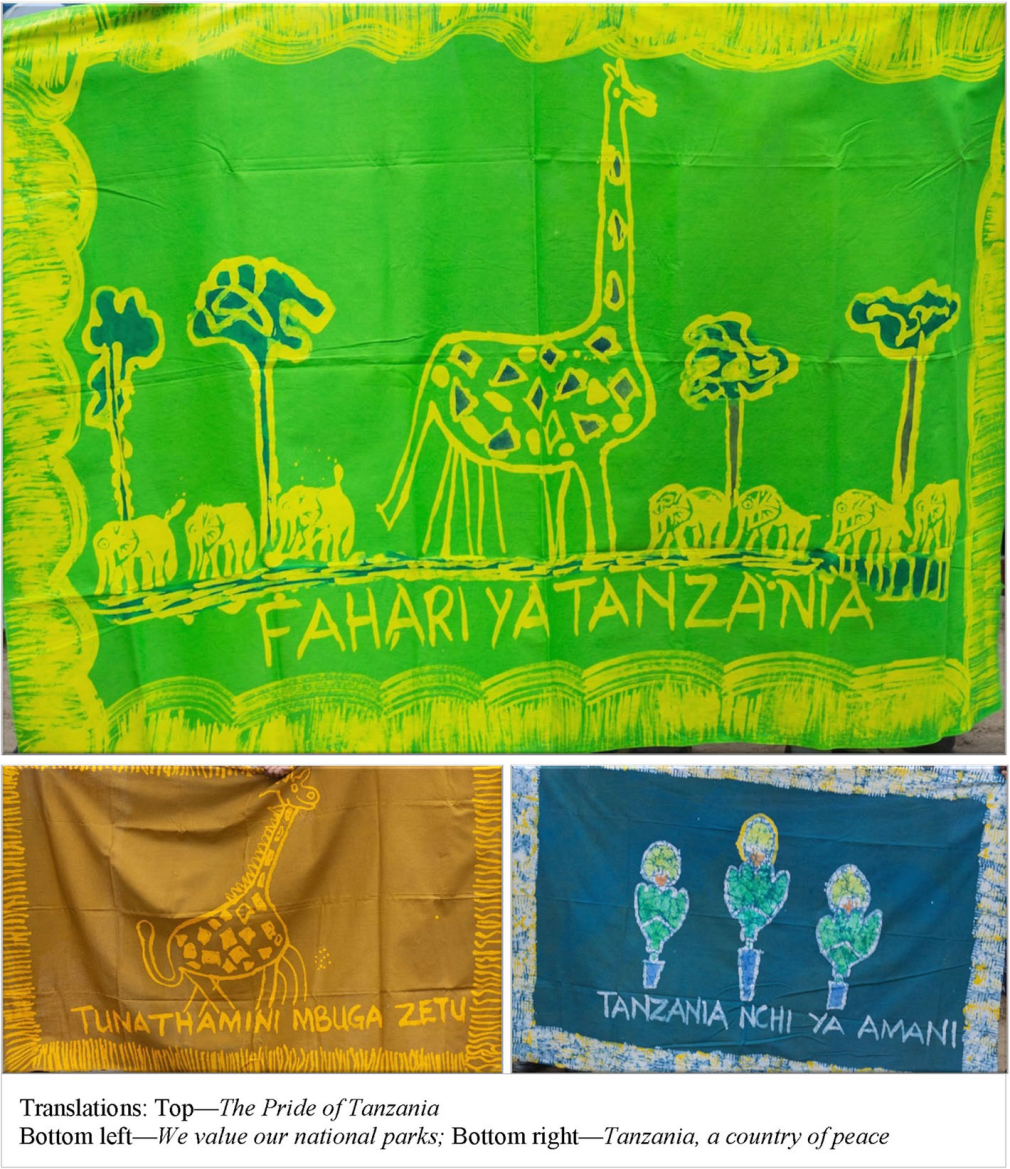
Adolescents designed Kangas printed on fabric

Involvement and contribution to the community was an important sub-theme. In particular the youth-led activity to create a community *Kanga* naturally bridged learning to the community. The kanga’s connection to historical and culturally grounded traditions in the community were conceived, designed and completed by *Discover* adolescents. Having a culturally relevant, community-oriented project allowed youth to draw connections between learning in the classroom and experiences in the community. Figure 2 represents an artifact of the Kanga designs that were printed on fabric and gifted to the community.

“Batiki was meaningful to me because we can make Batiki and it can bring development to the community. They can also teach people to read words which are written on the Batiki; once they read them, it can bring unity and love within the community.” (Girl, 10, 4^th^ Grade)

### Development of emotional mindsets and skills

Several participant responses focused on the importance of developing new learning mindsets and skills included in the content design of *Discover*. Parents/caregivers similarly expressed happiness with the ways that these new mindsets and skills provided their adolescent with a sense of purpose and motivated learning. This was reflected in responses from both parents/caregivers and adolescents.

“I started to observe initial changes because he (child) used not to be able to stand in front of the teacher and read a book, but these days he has confidence, even if he wants to do something, he will do it with confidence” (Mother, 40, Self-employed)

“She told me that, in the ‘purpose’ part of the project, she chose to be a lawyer, she asks me ‘dad is this lawyer's course worthwhile? And can it get us out of the pool of poverty?’ You see, I told her that she would make it; she would get money if she defended people legally” (Father, 36, Mechanic)

## Discussion

*Discover’s* theory of change posits that very early adolescence presents a window of opportunity to provide salient experiences of social emotional learning. *Discover* was designed to pair support for learning social emotional mindsets and skills with gender transformative content with active experiential learning practiced in mixed-gender small groups. Gender norms are shaped and reproduced by norms in institutions, cultural practices and social relationships and can directly or indirectly affect a multiple of health outcomes [45]. For example, a global systematic review conducted in 2006 found that gender stereotypes and different expectations about appropriate sexual behavior influenced sexual decision making [46]. This study found strikingly similar expectations of men’s and women’s behavior. Men were expected to be highly heterosexually active, and women chaste— women’s virginity at marriage often had high social value [46]. Matching gender transformative and social emotional learning opportunities to a sensitive window of development (ages 10 and 11) was particularly effective in this context because cultural norms lead to increasing gender segregation after sexual maturation. Parents/caregivers and community members endorsed mixed-gender group learning for VYAs. Additionally, targeting VYAs aligned with evidence from developmental science highlighting the unique opportunity for investment in early adolescence—before and during the onset of puberty—when the developing brain is most sensitive to social and emotional learning [47].

Moreover, the design of *Discover* included the hypothesis that the effects on social and emotional outcomes and gender equity outcomes would be amplified by opportunities to engage parents/caregivers and the community in the learning process. Bronfenbrenner’s ecological model of human development recognizes the importance of factors at the individual, microsystem (family and peer relationships), mesosystem (community) and macro level of influence in child and adolescent development outcomes [48]. While research in high-resource contexts provides evidence for the importance of parental engagement for positive adolescent development, less research has focused on the importance of parental engagement in low-resource contexts [49]. In addition, research programming for adolescents often excludes involvement of the community in intervention designs. Particularly in low-resource contexts in East Africa, community members serve an integral role in child and adolescent development [50–52].

*Discover* was designed to include parents/caregivers, teachers and community members in the intervention. Parental/caregiver and community involvement provided opportunities for active engagement in learning. These opportunities extended adolescent’s social emotional learning climate outside of program sessions. Research indicates that adolescent social emotional climate is important to reinforcing and supporting development of social emotional mindsets necessary to motivate use of social emotional skills [13]. Other programs in Tanzania have highlighted the importance of including adults and the community. The Vitu Newala Project in Tanzania focused on gender empowerment and provision of safe spaces for adolescent girls and results showed that in addition to empowering young female adolescents who participated, the gender norms, beliefs and behaviors of boys and adults in the community were also impacted [53, 54].

One of the most notable lessons learned from implementing *Discover,* was the effect on parental/caregiver and community members, through supported engagement and learning with adolescents. Several fathers involved in *Discover*, who are often overlooked in gender transformative programming, described shifts in their own gender norms, beliefs and behaviors. Fathers expressed recognition that their daughters needed time to study, rather than completing household chores. Fathers reported encouraging their daughter’s goals and enthusiasm for learning. One father described his motivation to change his own behavior and share household chores equally with his wife after using the parent-youth workbook and completing activities and reflective discussions with his child. These types of experiences are critically valuable for girls who often face harmful gender norms that limit their pursuit of heartfelt goals. Receiving reinforcement and recognition from parents/caregivers and at the same time, witnessing their parents/caregivers modeling those changes in their own gender norms, beliefs and behaviors can be extremely impactful at supporting gender transformative learning. Research indicates that it is possible to challenge dominant normative gender norms when masculinity and femininity is modeled in ways counter to inequitable gendered beliefs [55]. Parents/caregivers and extended family also described feeling closer to their adolescent through use of the workbook. Recognition of their child’s goals and dreams that contrast socially accepted norms can reinforce positive social and emotional development during a pivotal developmental window.

Another important theme that emerged from qualitative results, was the way *Discover’s* content appears to have transferred to siblings and peers through adolescent agency in communicating and practicing learning. Adolescents and their parents/caregivers described adolescents feeling empowered to actively share their learning with siblings and peers. This natural motivation to share learning has been shown in other gender transformative interventions. For example, a study found that boys were motivated to change their behavior after receiving appreciation from their sisters [56]. Similarly, in *Discover*, boys expressed the importance of sharing gender roles with their sisters and female peers. Parents explained that they had witnessed changes in boys, with boys taking on tasks usually relegated to girls.

Qualitative interviews also revealed the support and engagement offered by extended family and community members. Grandmothers, teachers and young adult facilitator inclusion provided a multi-generational reinforcement of adolescent learning. The community event was an important component of *Discover*, bringing together those who had participated in the community advisory board, teachers, parents/caregivers and extended family to recognize and praise the learning of adolescents. In addition, by providing creative means for adolescents to express their perspective in a tangible form (*the kanga),* a cultural artifact with deep meaning, adolescents were able to actively participate in bringing to fruition their own ideas while also demonstrating reverence for an important cultural tradition.

Consistent with [formative research results], adolescents, parents/caregivers and community members were enthusiastic about the use of technology for learning. Utilizing technology was a novel experience for most adolescents and offered a modality to support motivational learning, positive risk taking and natural curiosity in a stimulating social context. Through exploration of technology, adolescents expressed the highly arousing thrill of overcoming challenges and the social rewards associated with mastering skills in a collaborative learning environment. These positive learning experiences with technology will shape experiences in later adolescence as technology becomes increasingly pervasive.

These findings suggest that challenging gender inequities and norms through reflection, experiential learning and engagement with peers, parents/caregivers and community during very young adolescence has the potential to transform gender norms, beliefs and behaviors in the short term and could have a sustainable impact on longer-term health and well-being outcomes. Early adolescence is a uniquely opportune window of time to shape adolescent’s beliefs and behaviors towards more gender-equitable norms before social norms increasingly limit mixed-gender interactions. While social emotional learning is particularly important and naturally aligned with this window of development, experiential activities that allow adolescents to actively practice and reflect on social emotional learning in mixed gender groups can lead to powerful, salient experiences. These experiences are enhanced by opportunities to feel admired by peers, recognized for achievements and valued as equals. Research on the developmental science of adolescence has found that adolescents are particularly sensitive to and naturally motivated to achieve social rewards and admiration from peers [57, 58]. Adolescent programming that leverages the developmental sensitivity to social reward may enhance and reinforce social emotional learning.

Engagement with parents/caregivers and the community provides additional opportunities to model and reinforce learning. Our results indicate that parents/caregivers reported their own gender norms, beliefs and behaviors changing as a result of participation in *Discover.* Parents/caregivers play a critical role in challenging or perpetuating harmful gender norms. Girls are often burdened with an unequal share of household responsibilities in comparison to boys and these experiences can lead to social isolation that limit girls’ ability to explore their own interests. The potential of adolescents to themselves be changemakers in their parents/caregivers and communities gendered beliefs can help to challenge harmful gender norms and practices that pose risks to both girls’ and boys’ healthy development. Programs targeting early adolescence should capitalize on the opportunity to include gender transformative content during early adolescence. Introducing positive gender norms, beliefs and behaviors early in adolescent development can help reduce gendered health disparities that can amplify in later adolescence.

### Limitations

One limitation of this study was the difficulty in assessing the impact of the intervention on peers, siblings and community members not included in the qualitative study. In addition, this study conducted all interviews in Swahili, the native language, transcribed from audio and then translated to English. Data analysis was based on the translated scripts. It is possible that some meaning was lost during translation from Swahili to English. An attempt was made to control for this possibility by listening to the audio three times before transcribing, cross-checking the translations among the local research team and working with a data analyst who was a native Swahili speaker. While the local research team was trained in qualitative data collection methodology, there is a possibility of social desirability bias between the adolescent participants and interviewer or focus group moderator. However, both participant observations and in-depth interviews with parents/caregivers supported emergent themes from adolescent interviews and focus group discussions. This study was conducted in a peri-urban setting in Dar-es-Salaam Tanzania; therefore, the results may not be generalizable to other contexts. Despite these limitations, the findings from this study demonstrate the importance of parent/caregiver, peer and community engagement in gender transformation programs for very young adolescents.

## Conclusion

The findings of this study provide evidence of participant experiences with *Discover*, a social emotional learning intervention for very young adolescents. Study findings indicate changes in participant and parent/caregiver gender norms, beliefs and behaviors. Social emotional learning interventions that incorporate gender transformative content, and engage peers, parents/caregivers, and community members have the potential to take advantage of a unique window of opportunity in adolescent development that can amplify effectiveness of adolescent programing. Opportunities to leverage findings from adolescent developmental science can reinforce social emotional learning. Investment in very young adolescents can support positive developmental trajectories and empower adolescents to be agents of change towards more gender equitable communities.

## Supplementary Information

**Additional file 1.**

**Additional file 2.**

## Data Availability

The datasets generated and/or analyzed during the current study are not publicly available due to the sensitive age of the study participants (10–11-year-old) at baseline but are available from the corresponding author on reasonable request. The author will vet requests to be certain that appropriate IRB approvals and data safety guidelines are in place before distribution.

## Abbreviations

VYA: Very Young Adolescent
CAB: Community Advisory Board
NAB: National Advisory Board
