# Supplementary material for: Adolescent, caregiver and community experiences with a gender transformative, social emotional learning intervention
Source: Int J Equity Health. 2021 Feb 3;20:55. doi: 10.1186/s12939-021-01395-5 (PMC7860221; doi:10.1186/s12939-021-01395-5)
Supplement: Supplementary file 1 — Additional file 1. [file 12939_2021_1395_MOESM1_ESM.docx]

**Discover Learning**

**- Phase 3: In-depth Interviews with Adolescents -**

*Note: In-depth interviews are based on a Grounded Theory methodology. Not all questions will be asked of all participants. In addition, some questions that do not appear on this list may be included if relevant to the course of the interview. All questions will be within the scope of the interview topic, and no new questions that stray from the respective topic will be added to the interview guide without prior review and approval from OPHS.*

**Interviewer Name:**

Date: __/__/__

Interview start time __:__ AM/PM

Location:

**Demographic questions**

1. Are you…
   1. Male
   2. Female
2. How old are you?

______ years

**Purpose of the interview**

Hi*,* my name is _____ and I work with *Discover,* a project based here with partners from Save the Children, the Ministries of Health and Education – Tanzania and University of California Berkeley. Thank you for making time to meet with us. We are talking to you today to ask about you experience in *Discover,* ways you learn, how you interact with peers, schools, teachers and families, and, opportunities to improve your educational experiences using technology.

First, I'd like to ask you some questions about a typical day when you have school.

**Discover Learning**

1. What was your favorite game you played during Discover? Tell me about it.
   - What did you like about it?
   - What did you learn from playing this game?
2. Could you tell me about the mind mapping activity you did in the Discover project?
   - - What did you like about this activity?
     - What did you learn about your community?
3. Please tell me about what you thought about the scavenger hunt you did in Discover.

- What was challenging about the scavenger hunt?
- What was fun about the scavenger hunt?

1. What did you think about the create a Kanga activity?

- Was it easy to come up with a design with your team?
- What did the Kanga mean to you?
- What was it like to share the Kanga with your team?

1. What was your least favorite activity you did in the Discover project?
   - What didn't you like about it?
2. What is something new you learned during Discover?

- How will this be useful or important in your life?

1. Did you work on the parent-youth workbook with you caregiver?

- What was fun about the workbook?
- What was it like working with your parent/caregiver?

1. Did you share anything you learned from Discover with your siblings, parents or friends?
   - Tell me about what you shared

**Technology**

1. How did you like using tablets? (Probe for examples)

- What did you use the tablets to do?
- How was the experience?

1. What was the most difficult thing about learning how to use tablets?
   - How did you overcome challenges you faced?
2. What was the most fun or rewarding thing about using tablets?
   - Tell me about something fun you did with the tablets
   - What did you learn from playing the computer games ?
3. What was the most interesting thing you learned watching the Ubongo kids episodes and discussing them as a group??
   - Can you tell me something else you've learned from Ubongo kids?
4. What is your favorite Ubongo Kids character and why?
5. How do you think tablets could be useful in your future?
6. How did you like solving problems as a team using the tablet?
   - What was challenging?
   - What made it fun?

**Social Relationships and Learning**

1. How did you like working with your classmates during Discover?

- Was it different from how you learn normally with classmates? How?

1. Tell me about a time that you had to work with your friends or classmates during Discover.

- What did you have to do together?
- What worked well?

1. What was hard about working in groups? Tell me about it.

- How did you overcome the challenges you faced while working in groups?
- Did you like working in groups with both boys and girls? Why or why not?

1. Did you speak up when you had ideas in your group? Tell me about it.
2. When does working in groups / teams work well?
   - What type of project is best for teamwork? Can you give me examples of such projects that you did in Discover?
   - What size team works best?
3. Tell me about a time that your opinion or ideas helped the group solve a problem (probe for specific scenarios).
   - What made that work well?
   - What made it hard?
4. How did the facilitators help you to learn during Discover?
   - What makes him/her a good teacher?
   - What kind of things do they say or do that you like?

**Identity**

1. What does it mean to you to be a curious student?

- Can you give me an example?
- Can you give me an example of when you were curious?

1. What does it mean to you to be creative?

- Can you give me an example?
- Can you give me an example of when you were creative?

1. What does it mean to you to be a student who is persistent?
   - Can you give me an example?
   - Can you give me an example of when you were persistent?
2. Tell me about a time that you acted as a leader in a group.
   - How did you feel as the leader?
   - What was hard?
   - What else did you learn from this responsibility?
3. What does it mean to show generosity?

- Did you do anything during Discover that showed generosity?

**Close of interview:**

Do you have any questions or last comments for me?

Thank you very much for your time.

Interview end time: ___:___AM/PM
