# Supplementary material for: Adolescent, caregiver and community experiences with a gender transformative, social emotional learning intervention
Source: Int J Equity Health. 2021 Feb 3;20:55. doi: 10.1186/s12939-021-01395-5 (PMC7860221; doi:10.1186/s12939-021-01395-5)
Supplement: Supplementary file 2 — Additional file 2. [file 12939_2021_1395_MOESM2_ESM.docx]

**Discover Learning**

**- Phase 3: Interview with Parents after Intervention -**

**Interviewer Name:**

Date: / /

Interview start time : AM/PM Location:

Interview Identification Number:

Group (circle one): A B C

**Demographic questions**

Gender

1. Male
2. Female

How old are you?

What is your occupation? [please write occupation]:

What is your highest level of education?

- 1. No education
  2. Some Primary
  3. Completed Primary
  4. Secondary or higher

What types of relationships do you have with youth 10-11 years old? (Check all that apply)

- - 1. Parent
    2. Teacher
    3. Community leader
    4. School administrator
    5. Youth group worker
    6. Other :

**Interviews with Parents – After Intervention**

1. How well do you think the Discover Learning Program was implemented?
   1. What worked well?
   2. What could have been better?
2. In what ways, if any, has the Discover Learning program changed the way your child learns?
3. In what ways, if any, has the Discover Learning program changed the way your child takes advice from friends? From adults?
4. What changes, if any, have you noticed in your child’s ability to solve problems or challenges he/she faces?
5. If you have noticed any changes in your child, what are the positive ways it has influenced your child? What are the negative ways it has influenced your child? When did you start to see these changes?
6. What are the most important parts of the Discover Learning program?
   1. What makes these parts important?
7. What are the main challenges or weakness about the Discover Learning program?
   1. How did these challenges affect your child?
8. What suggestions would you have for improving this program?
9. In what ways, if any, do you think that Discover Learning may have changed the way your child learns at school?
10. In what ways, if any, has this changed your child’s knowledge about technology.
11. What do you think was the most significant change in your child as a result of being in the Discover Learning program?
12. Were you able to do the workbook activities with your child?
    1. If yes, what worked well?
    2. If not, what were the challenges to completing this workbook?
13. How important did you find the parent-child workbook to be in this program? What makes it important?
14. Describe an activity in the workbook that you enjoyed doing.
15. How did the workbook help inform you about what your child is learning during the sessions? How was this useful to you?
16. In what ways, if any, has using the workbook changed your relationship with your child?
17. In what ways, if any, has using the workbook changed how you interact with your child?
18. How would you describe Discover Learning to a parent at another school where the program was going to be implemented? What advice would you give them if they asked you if their child should participate?
19. What else you would like to share with me about your child’s experience in the program?
